# **The importance of histologic parameters of lacteal involvement in cases of canine lymphoplasmacytic enteritis**

**Published:** 2015

**Authors:** Giacomo Rossi, Matteo Cerquetella, Elisabetta Antonelli, Graziano Pengo, Gian Enrico Magi, Vincenzo Villanacci, Mohammad Rostami-Nejad, Andrea Spaterna, Gabrio Bassotti

**Affiliations:** 1 *School of Biosciences and Veterinary Medicine, University of Camerino, 62024 Matelica (Macerata), Italy*; 2 *Gastroenterology Unit, Perugia General Hospital, 06156 Perugia, Italy*; 3 *Veterinary practitioner, S. Antonio Clinic, 26020 Madignano (Cremona), Italy*; 4 *Second Pathology Section, Spedali Civili, 25100 Brescia, Italy*; 5 *Gastroenterology and Liver Diseases Research Center, Shahid Beheshti University of Medical Sciences, Tehran, Iran*; 6 *Gastroenterology Section, Department of Medicine, University of Perugia Medical School, 06156 Perugia, Italy*

**Keywords:** Dogs, Histology, Intestine, Lymphangiectasia, Lymphoplasmacytic enteritis

## Abstract

**Background**: The most frequent form of inflammatory bowel disease (IBD) in dogs is represented histologically, by lymphoplasmacytic enteritis (LPE), a histological category, often associated with other morphologic alterations including lymphangiectasia (LE). However, literature data on this latter topic are quite scarce and have mostly been obtained in single reports or in small series.

**Aim**: We evaluated some morphologic parameters of intestinal villi and lacteals in a large cohort of dogs, and correlated them with serum albumin and cholesterol values.

**Patients and methods**: We investigated 136 dogs (94 with LPE, and 42 with gastrointestinal problems different from IBD) and analyzed their clinical, laboratory (albumin and cholesterol values), endoscopic, and histologic variables.

**Results**: The LPE group showed significantly impaired clinical, laboratory, endoscopic, and histologic variables compared to controls. Affected dogs showed significant correlations between canine inflammatory bowel disease activity index (CIBDAI) scores and endoscopic and histologic variables. Moreover, the grade of hematologic changes were strongly related to the intestinal histologic variables, in particular those concerning villous and lacteals morphology.

**Conclusion**: Dogs with LPE had intestinal histologic abnormalities (height, width, height/width ratio, calculated for both villi and lacteals), whose degree correlated with the severity of hypoalbuminemia and hypocholesterolemia. Evaluation of endoscopic and histologic variables in association to the clinical findings may reveal useful insights for the pathogenesis of LPE and, hopefully, might lead to more targeted therapeutic approaches.

## Introduction

Similarly to humans, dogs may be affected by chronic intestinal pathologies; among these, inflammatory bowel diseases (IBD) play a prominent role (1). IBD in dogs present some peculiar aspects compared to those seen in humans, especially from a histological point of view (2). Indeed, the most frequent histological form associated to IBD in dogs is lymphoplasmacytic enteritis (LPE) (3, 4). This histological category of enteritis, often associated with other morphological alterations (5), results frequently associated with lymphangiectasia (LE) (6), and protein-losing enteropathy (PLE) (7,8).

Lymphangiectasia in the dog is assumed to be an acquired disease, and its etiology is generally idiopathic (4). It may also result from any type of obstruction to lymph flow in the lacteals, mesenteric lymph vessels or nodes, most frequently secondary to inflammation. Venous hypertension as seen with congestive heart failure can also lead to impairment of lymph flow (4, 7, 9). Lacteal dilation is not the primary problem; however, as the lacteals in the villi dilate and enlarge, the normal absorption ability of the intestine is compromised (7). The subsequent effects of hypoproteinemia and hypoalbuminemia can then harm or potentially cause the death of the animal. Breeds most commonly reported to be affected by LE are the Basenji, the Norwegian Lundehund, Wheaten terriers, Yorkshire terriers, Shar-peis, and Rottweilers. There is no reported sex predisposition (7). 

It can be difficult to differentiate LE from other associated diseases or pathological entities that may occur simultaneously (7). Indeed, it could be diagnosed concurrently with an IBD, but it is uncertain whether one precedes the other or if they both may stem from the same pathogenetic process. It is debatable whether intestinal LE should be broken down into two categories: idiopathic versus LE secondary to inflammation. Whether the inflammatory process often observed with LE is primary and contributory, or if it is an associated but separate pathological process has not been determined yet.

However, data on this topic are relatively scarce, and have been mostly obtained from single case reports or from small patient series.

Currently, there are no objective pathological parameters that indicate a value of cut-off above which the diameter of the lacteals can be considered indicative of biochemically (serum albumin and cholesterol concentration), but not yet clinically relevant LE. Thus, the purpose of the present study was the evaluation of some morphological parameters of intestinal villi and lacteals, in association with some laboratory findings in a large cohort of dogs with LPE, with particular emphasis on the association between clinical, histologic, and hematologic findings, and their relationship with histologically detected LE. 

## Patients and Methods

Data from dogs investigated for suspected IBD, and with a diagnosis of LPE in the period January 2002-January 2009 were retrieved. Inclusion criteria were: compatible clinical findings (i.e., weight loss, persistent or recurrent vomiting and/or diarrhea (2, 10), exclusion of other possible causes of chronic enteritis including intestinal parasites (2,11,12), blood chemistry (see below), and with endoscopic investigation (see below) and histology showing LPE. Subjects with incomplete data were not included in the study.

**Clinical data**

The severity of clinical signs was assessed by the standard canine inflammatory bowel disease activity index (CIBDAI), represented by the sum of the score of six different clinical signs (attitude/activity, appetite, vomiting, stool consistency, stool frequency, and weight loss) (13).

**Laboratory studies**

Blood samples were collected from the cephalic vein and, after sampling, were transferred in both plain and EDTA-containing vials. For the purposes of the study only albumin and cholesterol values were assessed. These variables were analyzed by means of a laser counter (Sysmex XT2000iV, Sysmec Corporation, Kobe, Japan) validated in dogs (14,15), and by an automated spectrophotometer (Cobas Mira, RocheDiagnostics, Basel, Switzerland) with reagents provided by Real Diagnostics Srl (Viterbo, Italy). 

**Endoscopic studies**

**Figure 1:**
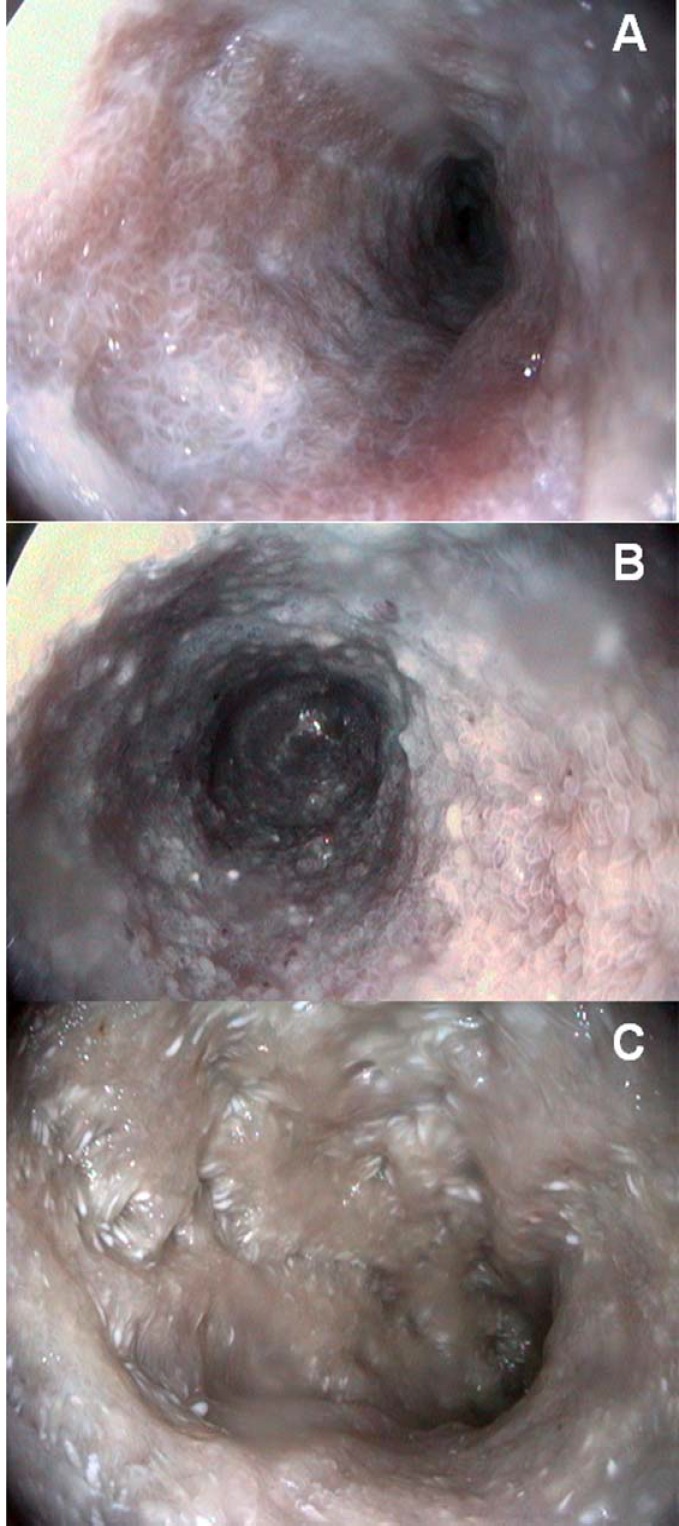
Endoscopic aspects of intestinal lymphangiectasia in dogs. Note the different degrees of severity characterized, in the early phase (A) by white villi with scattered white spots interspersed in an inflamed mucosa. In a more severe case (B) the milky aspect of the intestinal mucosa and the fluid content is diffuse, with edematous and well-evidenced villi. The advanced stage (C) characterized by many white spots and nodules, associated with submucosal elevation and a diffuse white aspect of the mucosa.

As a general procedure in our institution, to maximize the likelihood of visualizing LE, dogs (all IBD/LPE and non-IBD) undergoing endoscopy fasted for a minimum of 12 hours; then, 8-9 hours before the procedure they were fed a small fatty meal (using canned, not dry food, to which corn oil cream was mixed). Endoscopic procedures were carried out under general anesthesia (premedication with medetomidine, 2-3 µg/kg, induction with propofol, 2-2.4 mg/kg, and maintenance with a mixture of isofluorane/oxygen administered by an orotracheal tube). At least five images showing representative findings through the descending duodenum were obtained for every dog; these images were assessed blindly by one of the authors who did not perform endoscopic procedures. The findings were graded as mild, moderate and severe for each parameter (Figure 1), according to the scores for endoscopic parameters for animals proposed by the WSAVA gastrointestinal standardization group (16) (Table 1). At least 10 biopsy samples from the small intestine (duodenum) were obtained from each dog.

**Table 1:** Endoscopic variables and scoring according to the WSAVA Gastrointestinal Standardization Group (adapted from ref. 16)

Ability to distend the lumen
Edema
Hyperemia/vascularity
Discoloration
Friability
Texture
Hemorrhage
Erosion/ulcer
Lacteal dilatation
Contents

Score: 0= normal; 1= mild; 2= moderate; 3= severe

**Histological studies**

Immediately after sampling, intestinal biopsies were adhered to cellulose acetate paper and oriented in two ways: 5 longitudinally (submucosal side down and villi up on paper) and 5 perpendicularly (with the free side containing villi). Then, after fixation into iso-osmotic 10% buffered formalin, the specimens were embedded in paraffin maintaining the same orientation and cut to obtain 3µm thick sections, which were subsequently stained with hematoxylin-eosin.

Histologic analysis was carried out by an experienced pathologist, blinded to the clinical and endoscopic data, employing histologic criteria recently proposed by the WSAVA gastrointestinal standardization group for diagnosing gastrointestinal inflammation in dogs and cats (17). These criteria were implemented by a previously described adjunctive scoring system (18), used for a better definition of the concentration of inflammatory cells in intestinal inflamed areas and to evaluate the proportion of different types of leucocytes within the infiltrates. 

Inflammatory cells (neutrophils, mononucleate cells) and their organization in lymphoid aggregates were evaluated in 10 high-power fields (HPF, magnification x400). For inflammatory cells the following score (number of cells/HPF) was used: 0= <5 cells; 1= 5-10 cells; 2= 11-50 cells; 3= 51-100 cells; 4= >100 cells. Lymphoid aggregates were scored as: 0= absent and 1= present. For scoring inflammatory cells, leucocytes were quantified in different intestinal compartments (intestinal lamina propria, and epithelium of villus, basal crypt area, villus-crypt junction). All cellular types were calculated using a light microscope (Carl Zeiss Italia, Arese, Italy), a × 40 objective, a × 10 eyepiece, and a square eyepiece graticule (10 × 10 squares, with a total area of 62,500 μm^2^). 

**Figure 2:**
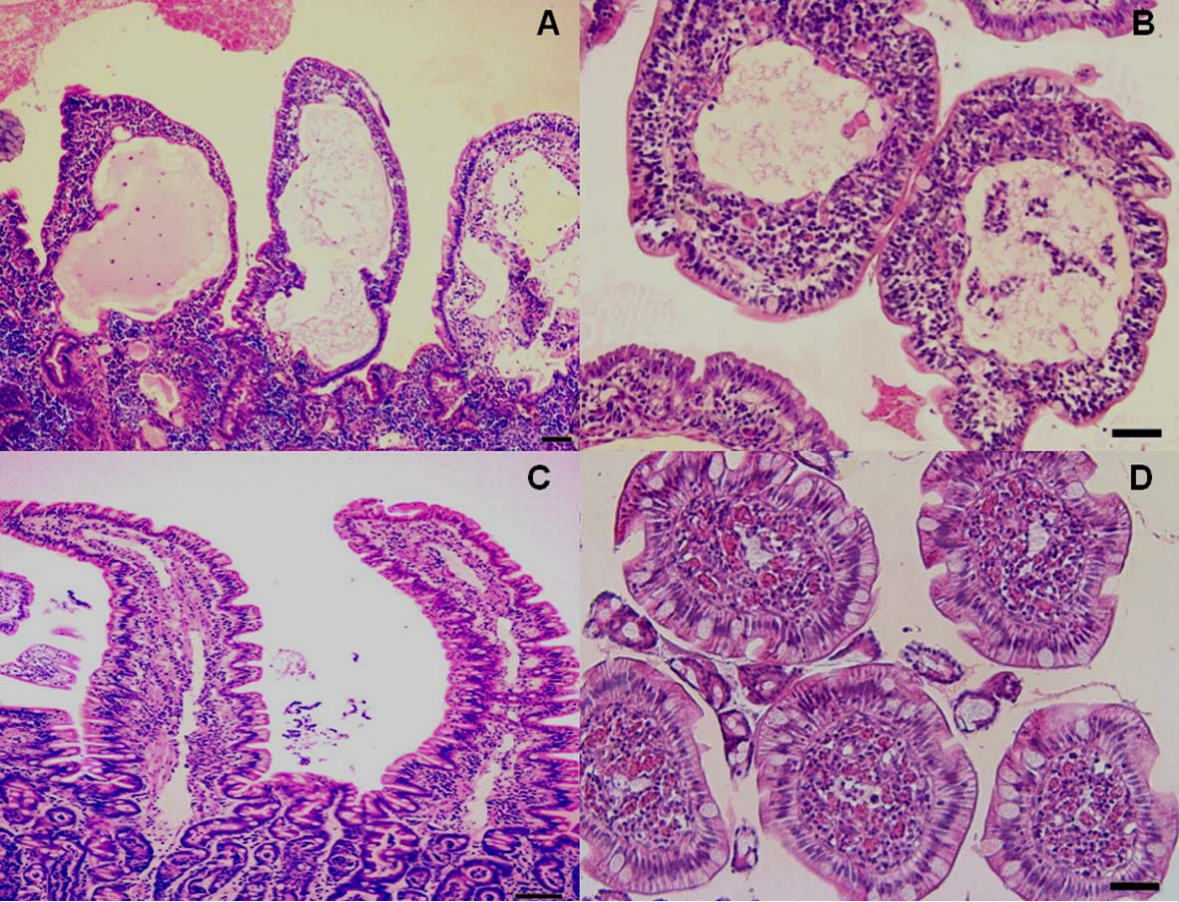
Histological aspects of intestinal lymphangiectasia in dogs (A and B) compared with normal findings (C and D). A. Numerous dilated and anastomosing lymphatic channels in the mucosa, containing mature lymphocytes, lined by flat epithelium and endothelial cells. Lacteal dilation distorts the villous architecture. A and B. The *lamina propria* shows engorged blood vessels and infiltration by chronic inflammatory cells, mainly lymphocytes and plasma cells. Abnormalities of villous height and width are evident in longitudinal (A) and coronal (B) sections. In normal mucosa (C and D) lacteals are small and sometimes difficult to observe in both longitudinal and coronal sections. H&E, scale bars 800 µm (A and C) and 200 µm (B and D).

At present, cases of lacteal dilation are evaluated as suggested by WSAVA criteria, and scored from 0 to 3 as follows: 0 = normal, when the central lacteal represented up to 25% of the villous lamina propria width on the longitudinal section; 1=mild dilation, when this width represents up to approximately 50%; 2= moderate dilation, when this width represented up to 75%; and 3 = marked dilation, when central lacteal represented up to 100% of the villous lamina propria (17). An aim of this study was to find morphological parameters by which to define in a more precise manner the values starting from which lacteal dilation can be considered, with regard to the criteria proposed by the WSAVA standardization group. Thus, we focused on the degree of correlation between the parameter of morphological dilation (height, width, height/width ratio, calculated for both villi and lacteals) and clinico-pathological findings (CIBDAI, endoscopic and histologic-inflammatory infiltrate-score, and albumin and cholesterol values). In this respect, the morphology of villi and lacteals (figure 2) was evaluated by measuring three randomly selected and morphologically well-preserved villi/ducts for each of the 10 (5 coronal and 5 longitudinal) sections. 

To calculate villous or lacteals height, the latter was obtained from the length from the tip of the villus or duct to the bottom, not including the crypt. Villi and lacteals width was calculated in both coronal and longitudinal sections. For the latter, the width was measured at about half the height of the villus. At this level, in fact, the diameter has a value intermediate between the diameter of the apex and the base of the villus/lacteal. These data were then compared with the measure of the width, obtained from the evaluation carried out on coronal sections. Both height and width values were obtained by means of an image analyzer (Nikon Cosmozone 1S7 CTR, Nikon Instruments SpA, Campo Bisenzio, Italy). Moreover, the height/width ratio (h/w) was calculated for both villi and lacteals.

**Controls**

This group was represented by dogs that in the same time period underwent endoscopic procedures and had laboratory screening, including assessment of albumin and cholesterol, always for signs related to gastrointestinal tract diseases, but diagnosed with foreign bodies (or suspected foreign bodies), esophageal dysfunctions, or acute-subacute gastritis or gastroenteritis (excluded from the diagnosis of IBD) necessitating of endoscopy. All dogs belonging to the control group received the same pre-endoscopical preparation described above for the study group, including the administration of a small fatty meal before endoscopy, since after this procedure also apparently healthy dogs can show small foci of lacteal dilatation (19).

**Statistical analysis**

Since values were not normally distributed, nonparametric tests were used for statistical analysis. Differences between patients and controls were assessed by means of the Mann-Whitney U test. Correlations between the various variables in LPE dogs were evaluated using the Spearman’s rank correlation coefficient. Values of p<0.05 were chosen for rejection of the null hypothesis. Data are presented as medians (95% CI). 

**Ethical considerations**

All dogs were evaluated in accordance with protocols approved by the University of Camerino Institutional Animal Care and Use Committee. The University of Camerino institutional review board approved the routinely used clinical protocol in both IBD/LPE and non-IBD animals. According to the guidelines of the Institutional Ethical Committee of University of Camerino (Comitato Etico Tutela Animali – CETA) it was not necessary to require formal approval from the CETA, since all dogs were studied under the owner’s written informed consent, for diagnostic or check-up purposes.

## Results

Overall, complete data from 136 dogs (94 with IBD/LPE and 42 non-IBD) were available for the study. Table 2 summarizes the results obtained in LPE and non-IBD dogs. The LPE group showed significantly impaired clinical, laboratory, endoscopic, and histological variables compared to non-IBD dogs. In LPE dogs, not all patients presented the same alterations of variables considered; in particular, not all were hypoalbuminemic.

All LPE dogs showed lacteal dilation (moderate to severe) at endoscopy, compared with 36% of mild findings found in the non-IBD group.

Concerning the various variables in LPE dogs: a) CIBDAI score significantly correlated with both endoscopic (rho= 0.67, p<0.0001) and histological (rho= 0.68, p<0.0001) score; b) there was a strong correlation between endoscopic and histological scores (rho= 0.83, p<0.0001); c) there was an inverse relationship between the histological score and albumin (rho= -24, p=0.017) and cholesterol (rho= -0.43, p<0.0001) levels; d) the height of the intestinal villi inversely correlated with albumin (rho= -0.23, p=0.025) but not with cholesterol (rho= -0.03, p=0.8) levels; e) the width of intestinal villi did not correlate with albumin (rho= 0.17, p=0.1) and cholesterol (rho= 0.002, p=0.98) levels; f) the height of lacteals was inversely correlated with albumin (rho= -0.23, p=0.03) but not with cholesterol (rho= -0.04, p=0.7) levels; g) the width of the lacteals was inversely correlated with both albumin (rho= -0.73, p<0.0001) and cholesterol (rho= -0.65, p<0.0001) levels; h) the villous h/w ratio was inversely correlated with albumin (rho= -0.24, p=0.018), but not with cholesterol (rho= -0.004, p= 0.9) levels; i) the lacteals h/w ratio was significantly correlated with albumin and cholesterol levels (rho= 0.6, p<0.0001 for both variables).

## Discussion

This study, conducted on a large sample of dogs with LPE showed that affected animals show several peculiar clinical and pathological features compared to non-IBD dogs. These features may be of interest in diagnostic and therapeutic approaches to the condition. Several studies have evaluated the normal canine and feline duodenal mucosa with Haematoxylin & Eosin and immunohistochemical staining, and showed that the normal villus length for an adult dog is 722 ± 170 µm, the normal crypt depth is 1.279 ± 203 µm, and the normal villus to crypt ratio is 0.7 ± 0.3 (20,21). A scoring system for lacteal dilation has also been suggested (17), but more precise reference values above which to consider dilated both villi and lacteals are still lacking.

In our series lacteal dilatation (lymphangiectasia) was frequent, even though it was not always clinically suspected, considering that clinical signs are highly variable, and diarrhea, the most frequent clinical sign, is common to many other diseases. In hypoalbuminemia was not present; however, even though this finding is suggestive of lymphangiectasia, it does not exclude the disease. Clinically, dogs included in this study presented symptoms in different association with each other; the classical signs of lymphangiectasia (i.e., ascites, edema, and hydrothorax) were observed only in a few cases, which showed severe morphological abnormalities of the intestinal mucosa and profound hypoalbuminemia.

**Table 2:** Clinical, endoscopic, and histological variables observed in IBD/LPE and non-IBD dogs. Data are presented as median (95% CI).

	LPE (n= 94)	non-IBD (n= 42)
Males/Females (%)	51/43 (54.2/45.7)	23/19 (54.7/45.2)°
Age (yr)	7.2 (7-8)	5.2 (3-8)°°
CIBDAI	5 (4-7)	1 (1-2)*
Albumin (2.7-4.4 g/dL)	2.7 (2.3-2.9)	3.4 (3-3.5)**
Cholesterol (129-148 mg/dL)	138 (127-145)	166 (148-195)*
Endoscopic score	21 (19-22)	0 (0-1)*
Histologic score	2 (2-3)	0 (0-0.8)*
Villous height (µm)	610 (613-659)	795 (724-831)*
Villous width (µm)	273 (261-290)	153 (136-171)*
Lacteals height (µm)	532 (517-582)	604 (598-689)*
Lacteals width (µm)	256 (245-262)	121 (119-225)*
Villous h/w	2.3 (2-2.5)	5 (4.5-5.6)*
Lacteals h/w	2 (1-2.5)	5 (1-6)*

° p= 0.89 compared to LPE;

°° p= 0.29 compared to LPE;

* p<0.0001 compared to LPE;

** p= 0.005 compared to LPE

All of the variables investigated here in LPE dogs were significantly different from control animals. In particular, the endoscopic and histological scores showed the greatest differences. 

With respect to clinical variables, higher CIBDAI scores suggested worse endoscopic and histological outcomes, as shown by a significant statistical correlation (Table 2). However, there is recent evidence that the endoscopic aspect alone may not be sufficient in this setting, especially concerning intestinal LE (9), and that other data (such as histological assessment) are needed. Multiple endoscopic variables were taken into consideration in LPE dogs, according to standard criteria (16), and the endoscopic score was compared to the clinical and histological ones, as well as to laboratory variables suggesting protein-losing (albumin and cholesterol) enteropathy. Thus, a direct correlation was found between the clinical variables (CIBDAI) and both endoscopic and histological scores, suggesting that a high CIBDAI may herald severe endoscopic and histological damage. Moreover, a worse histological score suggests lower levels of albumin and cholesterol; in particular, this may be due to damaged intestinal villi and lacteals, whose involvement was significantly related to albumin and cholesterol loss. Interestingly, lacteals wider than 108 µm were constantly associated with a severe (<2g/dL) loss of albumin. According to our observations, when the average value of the h/w ratio is below of 2.3 µm for villi and 10 µm for lacteals respectively, it is reasonable to consider already a condition of lacteal dilation, even if the values of albumin and cholesterol are still normal.

Also, we want to stress the importance of excluding secondary causes of lymphangiectasia, especially those related to some parasites that, as in human beings, may be clinically insidious (22).

In conclusion, this large study in LPE dogs showed that hypoalbuminemia was correlated to intestinal histological abnormalities, and particularly to those related to the height and width of the villi and lacteals. These findings, together with other abnormalities (such as alterations of the intestinal barrier (19)) may be of importance in the pathogenesis of LPE and in the origin of signs. 

We feel that the above evaluations (villous height and width, chylous-ducts height and width, villous h/w ratio and chylous-ducts h/w ratio) could be helpful when dogs presenting clinical sings but not hypoalbuminemia and hypocholesterolemia are diagnosed histologically with “mild dilated” (score 1) or even “normal” (score 0) lacteals. It will be interesting to establish whether, in such cases, followed over time, the histologic evaluation (also taking into account the above variables), could predict a possible future development of clinically evident lymphangiectasia. Of course, to further validate the parameters we investigated, comparative studies with the criteria previously proposed by the WSAVA standardization group (17) would be needed.

In our opinion, these data may be important not only for a better understanding and treatment of the veterinary pathology but also, as shown in other conditions (23), be useful to increase knowledge of human diseases.
